# Sociotechnical imaginaries in academic medicine strategic planning: a document analysis

**DOI:** 10.1007/s10459-024-10339-x

**Published:** 2024-05-27

**Authors:** Paula Rowland, Madison Brydges, Kulamakan (Mahan) Kulasegaram

**Affiliations:** Temerty Faculty of Medicine, University of Toronto, Toronto, Canada

**Keywords:** Discourse, Digital health, Sociotechnical imaginaries, Document analysis, Strategic planning

## Abstract

Purpose Along with other industries, healthcare is becoming increasingly digitized. Our study explores how the field of academic medicine is preparing for this digital future. Method Active strategic plans available in English were collected from faculties of medicine in Canada (*n* = 14), departments in medical schools (*n* = 17), academic health science centres (*n* = 23) and associated research institutes (*n* = 5). In total, 59 strategic plans were subjected to a practice-oriented form of document analysis, informed by the concept of sociotechnical imaginaries. Results On the one hand, digital health is discursively treated as a continuation of the academic medicine vision, with expansions of physician competencies and of research institutes contributions. These imaginaries do not necessarily disrupt the field of academic medicine as currently configured. On the other hand, there is a vision of digital health pursuing a robust sociotechnical future with transformative implications for how care is conducted, what forms of knowledge are prioritized, how patients and patienthood will be understood, and how data work will be distributed. This imaginary may destabilize existing distributions of knowledge and power. Conclusions Looking through the lens of sociotechnical imaginaries, this study illuminates strategic plans as framing desirable futures, directing attention towards specific ways of understanding problems of healthcare, and mobilizing the resources to knit together social and technical systems in ways that bring these visions to fruition. There are bound to be tensions as these sociotechnical imaginaries are translated into material realities. Many of those tensions and their attempted resolutions will have direct implications for the expectations of health professional graduates, the nature of clinical learning environments, and future relationships with patients. Sociology of digital health and science and technology studies can provide useful insights to guide leaders in academic medicine shaping these digital futures.

## Introduction

The future of healthcare is digital. This declaration graces the headlines of various reports, white papers, promotional materials, and fundraising campaigns. Whether this digital future will be a continuation of academic medicine as currently constructed, or whether these imagined futures suggest something far more transformational, is up for debate. With this paper, we invite a discussion on the future of academic medicine within the context of increasingly digitized healthcare systems. We accomplish this through a document analysis of current strategic plans produced by medical schools and academic health sciences centres in Canada. Through this analysis, we illuminate the various discourses operating in strategic planning of these key organizations, attending to the discourses that are most prevalent and those that are more marginalized. Our aim through this analysis is to explore dominant visions of digital health, how these visions are being distributed, and possible implications for the field of academic medicine.

Part of the challenge in understanding the potential and actual implications of digital health within the field of academic medicine lies in definitional ambiguities. In this paper, we will locate our study in broader discussions about (a) definitions of academic medicine as a strategic field and (b) definitions of digital health. Following this definitional work, we introduce the concept of sociotechnical imaginaries as a theoretical lens by which to explore visions of digital health and their effects. The concept of sociotechnical imaginaries draws together substantive bodies of literature from cultural and political studies with dynamics of sociotechnical systems as explored in science and technology studies (Jasanoff, 2015a). It is through this theoretical lens that our document analysis of Canadian strategic plans becomes conceptually transferable, as we identify visions of digital health that can be explored in global contexts. As we report on the findings from our document analysis, we illuminate multiple visions of digital health and their distributions. Our discussion explores possible implications for academic medicine as a strategic field navigating potentially transformative change. This study adds to the field of academic medicine by introducing the theoretically generative concept of sociotechnical imaginaries and inviting a foundation for further reflection, attentive research, and mindful leadership during eras of profound change.

### Academic medicine as strategic action field

A broad, global definition of academic medicine identifies the field as pursuing three aims: educating the next generation of physicians and scientists, researching causes and cures of diseases, and providing patient care (Katner, 2008). Some advocate to expand this triple mission to include a fourth imperative: social accountability (Smitherman et al., 2019). However, the way these missions are taken up and operationalized depend on the context, with substantive differences internationally (Ovseiko et al., 2010) and even within countries (Reich, 2014). Furthermore, while these three (or four) missions align at the broad level of an aspirational organizational vision, the day-to-day integration of multiple missions can be problematic for clinicians and educators experiencing missions as competing for time, resources, and legitimacy (Gonzalo et al., 2021; Hoff et al., 2006). On a broader level, the integrative aspirations of academic medicine are under pressure in the face of dwindling research funding, increased clinical demands, and legislative contexts that permit rather than mandate educational missions (Chhabra et al., 2022). In short, the vision of academic medicine is aspirational and far reaching. However, the operationalization of this vision is politically, socially, and organizationally complex.

The concept of strategic action fields (Fligstein & McAdam, 2012) is one way to grapple with the complexity of academic medicine. Fligstein and McAdam (2012) define a strategic action field as “a constructed mesolevel social order in which social actors (who can be individual or collective) are attuned to and interact with one another on the basis of shared (which is not to say consensual) understandings about the purpose of the field, relationships to others in the field (including who has power and why), and the rules governing legitimate action in the field” (p. 9). In this framing, the strategic action field is the subjectively shared *social* structure whereas formal organizations are the “objective entities in the world, with clear boundaries and legal designations” (Fligstein & McAdam, 2012, p. 64). With the focus on social dynamics of stability and of change, the concept of strategic action fields draws attention to ways organizations and individuals might collaborate with others, but also how they might compete and vie for advantage in their shared field (Fligstein & McAdam, 2012), particularly as they respond to internal and external challenges.

Drawing from theories in sociology, organizational studies, and social movements, the concept of strategic action fields shares intellectual alliances with other theories of fields (Bourdieu, 2005), institutional logics (DiMaggio & Powell, 1983; Scott, 2005), and broad theories of social movements (Fligstein & McAdam, 2012). The theory of strategic action fields stands apart from these other theories by drawing attention to the ways fields are constructed, how they might remain stable, and how they might change. Furthermore, in considering strategic actions fields as mesolevel social structures constructed through shared understandings and meaning-making of the purpose (and the rules) of the field, there is a distinction made between the concept of field and the organizations that make up the field. Key elements of the theory of strategic action field include: the definition of strategic action fields; a focus on positions in the field including the incumbents, the challengers, and the role of governance units; attention to social skill of actors; attention to the broader field environment; the role of outside shocks in shaping fields; and processes of field settlement leading to (temporary) stability (Fligstein & McAdam, 2012).

Applied to our interests in this paper, we conceptualize academic medicine as a strategic action field, united by a shared understanding of the tripartite mission of advancing education, research, and practice. In Canada, the formal organizations that comprise this field include universities, research institutes, and health service organizations. Of course, there are other organizations that comprise this field, but we limit our attention to these three for this paper. Even within these three types of organizations, there is much variation with different kinds of organizational and legislative relationships created between universities, research institutes, and health service organizations. The designation of academic health science centre (AHSC) is particularly relevant to our study, referring to a health service organization that includes the “teaching hospital” but might also include community-based health services and broader care networks. By conceptualizing academic medicine as a strategic action field, we direct our attention to the various organizations within the field, anticipate dynamics of both cooperation and competition within the field, and are attuned to destabilizing dynamics that might shift the rules and the distributions of power that have been previously settled. It is through this framing that we have become interested in the broader claims that digital health will transform health and healthcare as we know it. We are interested in how the field of academic medicine is responding to these bold visions of digital health.

### The future of healthcare is digital: conceptualizing digital health in academic medicine

As Henwood and Marent comment (2019), “digital health is both easy and hard to define” (p. 1). While there is not yet consensus about what is included under the umbrella of digital health, most definitions include a wide range of tools used for telemedicine and virtual care. Another category of tools relates to health information communication technologies (e.g. electronic patient records, computerized provider order entry, web-based eHealth applications), collectively called *eHealth*. More than a collection of tools, the concept of eHealth brings together sets of disparate concepts, including health, technology, and commerce (Oh et al., 2005). Mobile technologies (e.g. data producing wearable technologies, remote monitoring technologies, health related applications of various mobile digital devices) are becoming increasingly prominent, collectively referred to as *mHealth* (Lupton, 2012). More recently, advances in data science have introduced new forms of algorithmic medicine, incorporating artificial intelligence (AI) and machine learning into the complex processes of diagnosis and prognosis (Marent & Henwood, 2023). In our study, we are interested in how academic medicine is orienting towards these digital futures, inclusive of telemedicine and virtual care, eHealth, mHealth, and AI/machine learning.

For the purposes of our study, we are particularly interested in what has not yet been fully explored in the academic medicine literature. While there is a body of work on the use of AI, machine learning, Big Data, and natural language processing within medical education (Alrassi et al., 2021; Fan et al., 2020; Hodges, 2018; James et al., 2021; Khurana, 2020; Russell et al., 2023), and a growing surge of editorials, commentaries, and studies on the potential implications for new competencies for practicing physicians (Johnston, 2018; Karnieli-Miller & Neufeld-Kroszynski, 2020; Wartman & Combs, 2018), there are few explorations of what these changes might mean for physician identity, forms of legitimate knowledge that will shape the profession, and potential reconfigurations of both accountability and control of professional work. Some exceptions exist in the academic medicine literature, primarily in the form of commentaries or perspective pieces raising questions about possible implications for the ways physicians think (Cooper & Rodman, 2023) and what new kinds of uncertainties AI-enabled healthcare will create (Harish et al., 2021). In contrast, explorations of identity implications are more prominent in the sociology of digital health and in science and technology studies in the domains of healthcare. Taken together these social science studies illuminate the various contradictory promises associated with digital health technologies, the ways these technologies potentially reconfigure forms of legitimate knowledge in healthcare interactions, and reconfigure dynamics of control and accountability in healthcare decision making spaces (Henwood & Marent, 2019). Social scientists argue for productive interactions between (a) science and technologies studies of the sociomaterialities of new technologies, (b) sociology of the professional dynamics of digitalization, and (c) medical literature on experiences of using digital health technologies (Carboni et al., 2022). It is through these kinds of interdisciplinary interactions that we might best grapple with the complexity associated with our current epoch of change.

## Conceptual framing: sociotechnical imaginaries

Our study builds upon this literature in the sociology of digital health and in science and technology studies to contribute to the growing discussion within academic medicine. Drawing from the social sciences, we approach this study through the concept of sociotechnical imaginaries. Here, we use the definition from Jasanoff (2015a), where sociotechnical imaginaries are “collectively held, institutionally stabilized, and publicly performed visions of desirable futures, animated by shared understandings of forms of social life and social order attainable through, and supportive of, advances in science and technology” (Jasanoff, 2015a, p. 4). This concept draws together two substantive bodies of literature: (a) the construction of imaginaries as examined through cultural and political theory and (b) dynamics of sociotechnical systems as examined through science and technology studies (Jasanoff, 2015a). In this way, the concept of sociotechnical imaginaries becomes an analytical tool in the constructivist and interpretive social sciences, drawing attention to ways social worlds are made and unmade in relationship with prevailing ideas about science and technology. Attending to the ways imaginaries are made, how actors are enrolled in the collective vision, the ways these visions are potentially contested, and how these visions become institutionalized in material ways allows analysts to explore how actors may become oblivious to alternative forms of organization, order, and concepts of justice (Jasanoff, 2015b).

Methods associated with the concept of sociotechnical imaginaries tend to be qualitative, as researchers explore acts of meaning making. While research methods can include interviews and focus groups, documents are also a rich site to explore sociotechnical imaginaries. Consequential documents such as policies, legislation, and strategic plans are one site where “desirable futures (or … the monsters that policy seeks to keep at bay)” (Jasanoff, 2015a, p. 27) are made visible. A further strategy for making sociotechnical imaginaries visible is to deploy the use of comparisons, where comparing across social and political structures renders the content, context, and contradictions of alternative sociotechnical imaginaries available for analysis (Jasanoff, 2015a).

Having now introduced the concept of sociotechnical imaginaries, we can further elaborate on our aim for this paper. Through document analysis of strategic plans of the various organizations comprising the strategic field of academic medicine, we explore the predominate sociotechnical imaginaries shaping the field, how these imaginaries are being displayed in public facing documents, and what these imaginaries imply for professional knowledge and identity. By capturing this specific moment in time, our analysis sets the stage for future research on historical, geographical, and political comparisons.

## Methodology and methods

### Study design: document analysis sensitized by concept of sociotechnical imaginaries

Our overall study design is a form of document analysis. Here, we consider strategy documents as “crystallizations of strategic thought” (Vaara, 2015, p. 494) made available in the public domain. In addition, strategy documents serve to signal an organization’s preferred position in a broader institutional field (Fligstein & McAdam, 2012), indicating both belonging and differentiation (Tsoukas, 2018). We recognize that strategy documents are produced within a particular genre of writing, that they must be understood in relation to assumptions of common knowledge at that moment in time and place, and that they are material objects produced as part of a longer chain of texts. Finally, we recognize that strategy documents are not entirely instrumental, but also moral documents that reveal what the organization takes to be good practice (Tsoukas, 2018). While we recognize that strategy documents do not necessarily reflect what organizations ultimately do, we maintain that the analysis of strategy documents provides insight into the discourses shaping organizations and their conditions of possibility. In this way, we conceptualize documents as more than passive holders of information (Cleland et al., 2023) and instead engage with them as rhetorical devices with performative power.

In this study, we used the concept of sociotechnical imaginaries to direct our study design and analytical process. The concept directed our attention to strategic plans as consequential texts where sociotechnical imaginaries are articulated and made explicit. The concept also directed us to create an internal comparison within the broader field of academic medicine, looking to how leaders in (a) medical schools, (b) academic health science centres (AHSCs), and (c) health research institutes are imagining the digital futures of healthcare. Finally, we used sociotechnical imaginaries as a sensitizing concept in our document analysis, directing our line-by-line coding to include the use of metaphors, common tropes, and forms of interconnection between texts to illuminate the sociotechnical imaginaries deployed in the various texts.

### Study context

To provide context for our study, we first describe the organization of medical education in Canada. The academic training of physicians in Canada in 2023 takes place across 16 faculties of medicine/health sciences situated in universities and one independent school. All of these organizations host undergraduate and postgraduate training in medicine. Often other health professions also train within a faculty of medicine/health science. As learners transition across the continuum from undergraduate to postgraduate training, learning increasingly takes place in workplace settings. A large portion of clinical learning takes place in AHSC which have affiliation agreements with faculties of medicine while also having specific mandates for patient care and service provision. AHSCs have varying degrees of engagement with faculties of medicine with some academic centres having very close integration between staff and leadership (e.g. requirement for all staff physicians also to have faculty appointments and specific academic responsibilities; joint academic departments or centres with shared governance; common research ethics governance; shared buildings etc.) to looser affiliations where only some clinical departments or programs might be connected to a faculty of medicine. Of note, most AHSC are independent of universities and have their own mandates, budgets, and governing boards of directors. Given this separation, the strategic plans of AHSCs and of faculties of medicine are developed independently.

### Study procedures: collecting documents

We began with collecting current strategic plans, publicly available and published in English by faculties of medicine in Ontario, Canada (*n* = 6). We included all documents presented as the current strategic plan for the organization at the time of our search (June 2023). To provide additional context, we reviewed strategic plans from specific departments within one university’s faculty of medicine (*n* = 17). We then collected current strategic plans of AHSCs associated with these six faculties of medicine/health science (*n* = 23 plans) and research institutes associated with these AHSC (*n* = 5). In collecting this dataset, we included sites with full and partial affiliations with medical schools. To augment our analysis and challenge our early analytical thinking, we included additional faculties of medicine/health science across Canada who had posted a current strategic plan available in English (*n* = 8), creating a total of 14 strategic plans from faculties of medicine across Canada. Analyzing these additional plans provided reassurance that our findings were not a reflection of the unique idiosyncrasies of schools in one province, but instead accessed broader discourses at play in multiple schools across Canada. In total, we analyzed 59 strategic plans. Together, these 59 documents provided sufficient information power for analysis (Malterud et al., 2016). The determination of sufficient information power was made by (a) reflecting on the comprehensiveness of our sampling strategy and (b) confirmed through our ongoing abductive analysis that no new findings were generated through the inclusion of additional documents.

### Analytical strategy

Our analysis of the documents followed a practice-oriented approach (Asdal & Reinertsen, 2022), involving an iterative process of both content analysis (Hsieh & Shannon, 2005) and thematic analysis (Braun & Clarke, 2006). This involved successive readings of the documents (Bowen, 2009), first reading for immersion and general understanding, later engaging in close reading and examination of particular concepts, metaphors, and rhetorical turns, and final reading interpretively to attend to the rhetorical work of the documents themselves. This interpretive phase of the analysis involves line-by-line coding of the documents, illuminating assumptions that are being made, discourses that are being drawn upon, and noting discourses that are potentially being excluded (Rapley, 2007) or otherwise left unsaid (Presser, 2023). This phase of coding was both inductive and deductive, as we deployed the concept of sociotechnical imaginaries to direct our attention to metaphors and rhetorical turns while also remaining open to concepts as they are displayed in the documents themselves. Finally, we engaged in abductive analysis (Tavory & Timmermans, 2014, 2019), examining our emerging analysis in light of existing literature. In these ways our document analysis strategy is aligned with other methods in qualitative research, deploying a systematic procedure for selecting, reviewing, and evaluating texts in order to elicit meaning, gain understanding, and develop knowledge (Bowen, 2009). The outcome of our document analysis is a set of themes illuminating how the field of academic medicine is discursively orienting towards a digital healthcare future.

## Results

In this paper, we have opted to not identify any organization by name. Our intent is to analyze the various discourses available in the documents, not to invite comparisons across specific organizations. When we present data from medical schools, we provide some geographical detail (e.g. Med East, Med Ontario (ON), Med West) and a participant number to demonstrate the distribution of exemplar quotes across sites. Quotes from academic health science centres are referenced by category (e.g. AHSC) and a participant number. Documents from AHSCs included: strategic plans for AHSC providing broad services for the community and province as well as speciality care centres (e.g. cancer care, rehabilitation) and population specific care centres (e.g. pediatrics, elder care). Research institutes documents are indicated by the acronym “RI” and a participant number (e.g. RI1). In the sections that remain, we describe how organizations are strategically positioning themselves in relation to a “digital future of healthcare” and discursive differences across organizations. We present these findings as three themes: continuations, disruptions, and reimaginations.

### Continuations: digital health as an extension of existing dynamics of knowledge and practice

In a selection of the strategic plans, the future of digital health is presented as a continuation of existing dynamics of knowledge and practice. In this framing, digital health will expand the competencies of physicians and expand the possibilities of evidence-based research, but will not fundamentally disrupt the existing rules or distributions of power in the broader field of academic medicine. For example, while half (7/14) of the plans from medical schools acknowledged the role of technology to enhance existing educational practices and processes (e.g. “invest in new cutting-edge technology to help us do our work – virtual reality, new web platforms, video-interviewing and advanced communication platforms” (Med ON2), “optimize IT processes and strive to provide learning management systems that reflect state-of-the art technology” (Med ON3)), few (3/14) explicitly explored the possibly transformative implications of emerging technologies on clinical work. Three medical school plans identifying emerging technologies as an area of strategic attention homed in on digital technologies and data sciences. For example, one declared “a new, specific focus … on the strongly emerging and cross-cutting role of digital technologies and data sciences in current and future evolutions of health and clinical care” (Med East2; emphasis in original) while another identified the “increasing role of informatics, big data, analytics, and evidence-based practice” (Med ON3) as a key driver of change shaping today’s medical education. The third school was even more specific, identifying the need to “build capacity to reflect the emerging role of AI in health professions” (Med ON4). While other plans referred to new collaborations with “digital health and med-tech industry” (Med ON1), these three strategic plans stand apart from the others for their specificity. In contrast, all schools identified a commitment to prepare graduates for emerging futures, using language such as “empowering learners to meet evolving societal needs and career requirements” (Med West2). Presumably, emerging technologies are part of the landscape of “evolving societal needs” and within the scope of these strategies.

In a similar vein, research institutes heralded the power of increasingly datafied healthcare environments to advance the research mandate where “we will leverage the opportunity to learn from large and diverse groups of people. The insights, experiences, and biological and clinical data that we collect will be mined to test hypotheses and generate new ideas – feeding a learning health system to deliver patient-centred care” (RI1). Throughout the Research Institutes’ plans, there is a projected future where increased access to data would serve to strengthen and deepen the research enterprise. However, digital health was positioned as part of a broader movement towards “unleashing the power of technology and innovation” (RI1) without fundamentally shifting the distribution of powers or identities across the field of academic medicine.

### Potential disruptions: shifting sociotechnical imaginaries

Where the medical schools and research institutes tended to refer to technology somewhat generically, AHSCs were explicit. Information technologies, virtual technology-enabled home and community care, data sciences, and AI augmented decision making featured strongly in these plans. For example, one site declared “data and analytics must be woven into the fabric of our organization until it is automatic in our decision making” (AHSC ON15). Several plans identified digital health as a key strategic priority, often with a focus on implementing and optimizing electronic health records. These information technologies were declared to be “integral in supporting the best possible care” (AHSC ON2), related to improvements in “patient safety and efficiencies in clinical outcomes” (AHSC ON8), and foundational to the futures of “precision care” (AHSC ON13). With this moment in time declared as “an explosion in digital health innovation” (AHSC ON15), most of the AHSCs identified digital health as a key priority. Expressive prose included promises of “increasing adoption of AI and deep machine learning resulting in a future where patients, providers, and systems will harmoniously interact and data will continue to grow exponentially” (AHSC ON11). Where there were hints of caution in the AHSC’s strategic plans, these cautions were related to “safeguarding patient privacy” (AHSC ON18) and ensuring appropriate data governance (AHSC ON13).

Many of these AHSC documents identified the global health human resource crisis, shifting expectations of patients, and unsustainable funding models as key drivers shaping strategic planning. In 2020, the world-wide experience of the COVID-19 marked a substantive shift in the language and focus of strategic plans. Attention to dynamics of equity, diversity and inclusion featured in most AHSC plans authored before 2020. All plans authored after 2020 acknowledged the substantial disruptions associated with the COVID-19 pandemic and a growing awareness of social inequities characterizing Canadian healthcare. Where AHSC plans identified strategic activity in the domains of digital health and technology-enabled healthcare, these activities were positioned as a solution to almost all of the pressures identified as key drivers. Table 1 provides a list of exemplar quotes where digital health is foregrounded as a particular kind of solution by AHSCs. These quotes are distributed across speciality centres (e.g. cancer care, pediatric care), urban community hospitals, and rural health centres. In these exemplar quotes, digital health, virtual care, and data sciences are rhetorically packaged together as promises to optimize efficiency, expand the reach of healthcare services beyond geographical boundaries, and improve patient care experiences. Furthermore, the expansion of digital health, virtual care, and the increasing datafication of healthcare organization is presented as an integrating strategy appealing to the hopes and needs of multiple stakeholders: patients, administrators, policy makers, governments, and industry partners. Even allowing for the aspirational rhetoric that characterizes much strategic plan writing (Vaara, 2015), the general impression is one of “techno-utopia” (Lupton, 2014) where the goals of various stakeholders are seamlessly aligned and equally addressed by the promises of technological solutions to shared problems.

**Table 1:** Exemplar quotes from academic health science centres’ strategic plans

Quote	AHSC Identifier
We will derive meaningful insights from our rich data sources to optimize operations, inform care, fuel new research, and enhance learning	AHSC ON11
The strategic enabler … is critical to the organization’s financial health and future success, and includes expansion of digital health …	AHSC ON16
Drive improvement in care experiences by enhancing the quality of data and analytics	AHSC ON2
As a critical underpinning of (the strategy), we will integrate data assets across the (AHSC) enterprise to deliver value to patients and families, staff, partners, and governments	AHSC ON13
Modernize corporate technology to improve the integration and efficiency of core business functions	AHSC ON8
Our progression as an evidence-based, quantitative, insight driven organization is based on having well-presented, and easily understood data tightly integrated into our clinical operations and business processes	AHSC ON15
Together with our partners, we will enable delivery of care beyond our walls, through virtual care, system navigation, and creation of new pathways	AHSC ON14

### Reimaginations and reconfigurations

In the desirable futures primarily depicted by the AHSCs, there are several explicit and implicit reconfigurations. In some cases, the need to reimagine healthcare is stated baldly where one organization promises to “deliver a new health information system to reimagine the way we care for patients and learn from them” (AHSC ON18). The same organization declared that the new health information system would “rewire our collective brains” (AHSC ON18) and “change how you think and how you work” (AHSC ON18). In other plans, the emphasis on virtual care models and remote patient monitoring was heralded as “the future of healthcare, especially for (rural regions)” (AHSC ON21). Even in urban centres, there is a declared appetite to evolve ambulatory care along with virtual care models and data collection strategies in order “to help reduce reliance on hospital beds and space” (AHSC ON15). In this imagined future, there is a prominent role for patients where “our goal is to challenge today’s fragmented system and put consumer-friendly apps and tools in the hands of patient, families, and providers” (AHSC ON18). In addition to a more active, digitally-enabled role, patients are also positioned as rich sources of research insight and practice-innovation “with each patient now a big-data source” (Med East2). AHSCs, medical schools, and research institutes all identified new forms of knowledge and expertise required to capitalize on these data futures, inviting more collaborations with data scientists, engineers, and machine learning experts. In summary, there is very little that is left unchanged in these future imaginaries of the AHSC. Patient roles, models of care, optimal configurations of expertise, and physical spaces of hospitals are all potentially reimagined and reconfigured in these aspirational futures.

Notably, explicit references to “evidence” or “evidence-based practice” were largely absent in the dataset. Where there were exceptions, treatment of the concept of evidence was relatively brief, isolated to lines such as “enhance focus on and capacity for translational research to ensure discoveries make their way from bench to bedside and evidence makes its way into practice in a scalable manner” (Department of Medicine, ) and “strengthen evidence-informed practice, knowledge dissemination and innovations” (Department of Speech Language Pathology). In the absence of explicit use of the term “evidence” or “evidence-based practice”, we looked to related concepts of research, clinical trials, and clinical practice guidelines. Here, we found two trajectories. On the one hand, the increased digitization of healthcare was positioned as accelerating evidence generation progress, where one research institute declares “we will establish a digital platform for excellence in data collection, sharing, analysis, and reporting – providing the architecture to enable technological advances” (RI1). In these ways, advances in machine learning may very well enhance the research enterprise that underlies evidence-based medicine. On the other hand, much of the enthusiasm for AI in these AHSC strategic plans focus on the unique capacities of AI to “uncover previously undetectable trends” and “uncover unforeseen linkages by integrating diverse data sources to enhance the prevention, detection, diagnosis, and treatment” (AHSC 11). With the focus on health information systems, data mining, and “big data”, the impression is that improvement of healthcare will not come from looking to the published literature. Instead, strategic attention is directed towards data produced *within* AHSCs. This is a substantive shift in organizational attention, potentially marking a separation from the ways evidence-based medicine has been historically conceptualized and operationalized in these organizations.

## Discussion

### Absences or misalignments?

Through this analysis of strategic plans, we see potential absences in the rhetoric of medical schools and research institutes as compared to their AHSC counterparts. As outlined in our analysis, medical schools are embracing a technologically enhanced future. We do not mean to imply otherwise. Medical schools specifically outline the possibilities to enhance educational practices through various technologies, learning management systems, and the power of data analytics. There are mentions of adjusting curriculum and competency profiles to anticipate a future where clinical decision making is augmented by AI. In some cases, medical schools are explicit about digital futures and associated implications for health professions education. However, the strategic plans of medical schools largely under-specify the kind of transformations anticipated for Canadian healthcare. It is possible that the futures being anticipated by medical schools and AHSCs may differ by degree, rather than by substance. However, it is also possible that these imagined futures might become a site of struggle, with competing visions for the role of health professions and health professional knowledge in these digital futures. Early studies of professions and professional knowledge have under-estimated the influential role of clinical workplaces and employing organizations on professional knowledge, power, and identity (Evetts, 2013). Learning from past transformations of healthcare work, it seems wise to attend to these broader dynamics, particularly as academic medicine becomes influenced by new forms of knowledge, expertise, and power associated with data science, AI, and associated industry interests acting within health service organizations.

### Sociotechnical imaginaries and creating futures

While the medical schools and research institutes might be under-specifying potential transformations of Canadian healthcare, AHSCs are projecting a robust vision of healthcare systems and organizations transformed by digital health technologies. Included under this umbrella of digital health technologies are health information systems, virtual care technologies, remote monitoring technologies, and the potential for all forms of decision making to be augmented by machine learning capabilities. Organizations that have a longer history with concepts, tools, and strategies associated with precision medicine (e.g. cancer care centres) are the most specific about these possible futures and their current manifestations. However, the vision of digitally enabled solutions to healthcare’s most pressing problems also permeated the strategic plans of rural centres and community hospitals. In light of increasing digitization of healthcare, academic health science centres are strategically turning their attention *inwards* towards internal data collection, curation, and analysis capacities. Directing attention inwards may have implications for the knowledge regime of evidence based medicine, historically operationalized as practitioners and organizations looking *outwards* towards established bodies of literature (Rowland et al., 2022). Furthermore, the growing investment in various forms of technology involves shifting networks of expertise in these sites, inviting interdisciplinary teams to include data scientists and engineers. These strategic plans also seem to signal a further dissolution between the boundaries of providing care and generating research within academic health science centres. Whereas the teaching hospitals of the past required alignment of the patient experience to the teaching mandate (Rowland et al., 2019) (i.e. to be a patient in a teaching hospital required patients to become part of the broader clinical teaching “material”), current iterations may treat every patient encounter as an opportunity to generate data. This reordering of relationships with patients also has implications for care and learning practices.

Our analysis of strategic plans largely aligns with other studies of digital transformations of healthcare organizations, exploring how health service organizations are being reimagined and reconfigured through the promises of digital health (Gardner, 2022; Hoeyer, 2019) and growing entrepreneurial trajectories (French & Miller, 2012). For example, Hoeyer’s (2023) extensive ethnographic study of Denmark’s healthcare data infrastructures traces tensions that emerge when data is being produced, analyzed, and used in simultaneous attempts to produce knowledge, health, governance, and wealth. Each of these aims of data work are governed by different sets of values. Furthermore, the ways these values are pursued by different actors in the system have implications for the ways rights, risks, and responsibilities are distributed (and redistributed) across care work (McLoughlin et al., 2017). In this way, the transformation towards digital health is more than a translation of existing practices or the use of digital repositories to store clinical knowledge. Instead, these tools participate in the moral ordering of clinical work (McLoughlin et al., 2017), having implications for the ways care is provided, the clinical learning environment is organized, and what it means to be a competent professional. The result is a collection of paradoxes, where multiple but contradictory visions of current circumstances and future possibilities are held to be true. Considered in the context of the existing science and technology literature, our current study suggests the possibility for emerging tensions between the robust sociotechnical imaginaries being pursued by AHSC and the possible tensions clinicians might experience as their day-to-day work is transformed in these clinical sites.

### Implications for academic medicine

Looking through the lens of sociotechnical imaginaries, we see these strategic plans as framing desirable futures, directing attention towards specific ways of understanding problems of healthcare, and mobilizing the resources to knit together social and technical systems in ways that bring these imaginaries into fruition. However, academic medicine is a complex field, pursuing multiple mandates of patient care, research, and health professions education. In the face of such organizational complexity, there are bound to be tensions as these sociotechnical imaginaries are wrestled into material realities. Many of those tensions and their attempted resolutions will have direct implications for the expectations of health professional graduates, the nature of clinical learning environments, and relationships with patients. Some authors claim that the current moment in time reflects a transformation even larger than the shift to evidence-based practice (James et al., 2021), potentially challenging evidence-based medicine as the dominant knowledge regime (Kenny et al., 2021). Understanding how the linked ecologies (Abbott, 2005) of educational institutions, professional associations, and health service organizations are orienting towards a digital future should be a matter of interest for leaders in academic medicine.

In addition to considering implications for competency development and associated curricula, future research can draw upon robust social science traditions exploring the meanings, implications, and unintended consequences of these kinds of transformations. Working in collaboration with social scientists, leaders in academic medicine can draw upon the social science of quantification (Porter, 1996) and information infrastructures (Berg, 2001), sociology of professions (Abbott, 1988; Freidson, 1988; Larson, 1977/2013), and science and technology studies (e.g. Latour, 2007) to better understand the reconfigurations of these sociotechnical systems (Carboni et al., 2022) and their possible implications for academic medicine. There is also an opportunity to relate to broader technopolitics of this moment in time, as society writ large becomes increasingly datafied and digitized, potentially reframing our most fundamental human relationships (Spar, 2020). Much as a sociology of evidence-based practice has revealed the ways in which evidence-based medicine began as a particular kind of sociotechnical imaginary and evolved into a global web of institutions, experts, technologies, devices, and policies that define what healthcare is and what kinds of help we do (or do not) receive as patients (Broom & Adams, 2012), a sociology of digital health and the adjacent promises for digitally-enabled personalized medicine can help us see sociotechnical dynamics as they are evolving in the moment (Kenny et al., 2021). In the process, we may choose to question how digital work is being imagined, how it is being distributed, and to what uses it is being designed. The purpose here is not to dismantle these aspirational digital futures, but to contextualize, balance, and potentially emphasize voices that might become marginalized in these imagined futures. Arguably, the desired aim of academic medicine is not just become data-driven, but to become data wise (Hoeyer, 2023), an aspiration that is socially, politically, technically, and epistemologically complex.

### Limitations

We recognize the limits of analyzing strategic plans as particular kinds of texts. An absence in a plan does not necessarily equate to an absence in activity. Furthermore, practices of creating strategic plans are culturally and historically situated. Absences in the produced texts might reflect a style of writing and reporting, rather than an absence of organizational consideration. That being said, these strategic plans are in the public domain and serve as a declaration of organizational focus (Vaara, 2015). They also serve as a proxy for moral ordering, declaring what an organization indicates it *should* direct attention towards (Tsoukas, 2018). Given their role in directing organizational attention and identity, the analysis of strategic plans contributes to our understanding of the field of academic medicine. We further recognize the boundaries of our study. To maintain feasibility of the study and to ensure the trustworthiness of our document selection process, we focused primarily on one province in Canada. To elaborate our understanding, we expanded to included strategic medical school plans publicly available in English from across Canada. Rather than claiming that our results are generalizable to other contexts, our aim is to use the results of our study to display our theoretical concepts in ways that help others to appraise their own contexts with new insights (Merriam, 1988). Given the resonance of our findings with the various promises being made in the UK (Gardner, 2022; McLoughlin et al., 2017), Denmark (Hoeyer, 2023), and Australia (McLoughlin et al., 2017), we believe our analysis is tapping into broader promissory discourses about the role of technology in healthcare.

## Conclusions

In this study, we discursively examined the strategic plans of faculties of medicine/health sciences, academic health sciences centres, and health research institutes in Canada. Our aim was to better understand how the “digital revolution” shaping broader systems of work are understood, imagined, and deployed in the strategic action field of academic medicine. In order to better understand the effects of these discourses of technology sweeping through healthcare institutions, we draw upon the concept of sociotechnical imaginaries. In deploying this concept, we hope to contribute to the theoretical interrogations of healthcare transformations so that we may act wisely at various strategic choice points (Flyvbjerg, 2001). Current iterations of academic health science centres’ strategic planning point towards broader shifts in knowledge regimes that have implications for professional knowledge and identity, as well as constructions of what it means to be a patient. Further exploration of sociotechnical imaginaries of data-driven healthcare systems will allow us to explore the possible (and predictable) tensions of its operationalization and the associated implications for academic medicine. Drawing upon social science studies of data work in contemporary healthcare systems, we join others cautioning against acritical acceptance of data-driven imaginaries and instead hope for data-wise sociotechnical arrangements that support *health* and health *care* in increasingly digitalized environments (Marent & Henwood, 2023).

## Data Availability

No datasets were generated or analysed during the current study.

## References

[CR1] Abbott, A. (1988). *The system of professions: An essay on the division of expert labor*. University of Chicago Press.

[CR2] Abbott, A. (2005). Linked ecologies: States and universities as environments for professions. *Sociological Theory*, *23*(3), 245–274. 10.1111/j.0735-2751.2005.00253.x

[CR3] Alrassi, J., Katsufrakis, P. J., & Chandran, L. (2021). Technology can augment, but not replace, critical human skills needed for patient care. *Academic Medicine: Journal of the Association of American Medical Colleges*, *96*(1), 37–43. 10.1097/ACM.0000000000003733. 32910005 10.1097/ACM.0000000000003733

[CR4] Asdal, K., & Reinertsen, H. (2022). *Doing document analysis: A practice-oriented method*. Sage.

[CR5] Berg, M. (2001). Implementing information systems in health care organizations: Myths and challenges. *International Journal of Medical Informatics*, *64*(2), 143–156. 11734382 10.1016/S1386-5056(01)00200-3

[CR6] Bourdieu, P. (2005). The dynamics of the fields. In S. P. Hier (Ed.), *Contemporary sociological thought: Themes and theories*. Canadian Scholar’s.

[CR7] Bowen, G. A. (2009). Document analysis as a qualitative research method. *Qualitative Research Journal*, *9*(2), 27–40. 10.3316/QRJ0902027

[CR8] Braun, V., & Clarke, V. (2006). Using thematic analysis in psychology. *Qualitative Research in Psychology*, *3*(2), 77–101. 10.1191/1478088706qp063oa

[CR9] Broom, A., & Adams, J. (2012). A critical social science of evidence-based healthcare. In A. Brooms & J. Adams (Eds.), *Evidence-based healthcare in context: Critical social science perspectives*(pp. 1–19). Routeledge.

[CR10] Carboni, C., Wehrens, R., van der Veen, R., & de Bont, A. (2022). Conceptualizing the digitalization of healthcare work: A metaphor-based critical interpretive synthesis. *Social Science and Medicine*, *292*, 114572. 10.1016/j.socscimed.2021.114572. 34839086 10.1016/j.socscimed.2021.114572

[CR11] Chhabra, K. R., Diaz, A., Byrnes, M. E., Rajkumar, A., Yang, P., Dimick, J. B., & Nathan, H. (2022). Challenges and opportunities for the academic mission within expanding health systems: A qualitative study. *Annals of Surgery*, *275*(6), 1221–1228. 10.1097/SLA.0000000000004462. 33201110 10.1097/SLA.0000000000004462

[CR12] Cleland, J., MacLeod, A., & Ellaway, R. H. (2023). CARDA: Guiding document analyses in health professions education research. *Medical Education*, *57*(5), 406–417. 10.1111/medu.14964. 36308050 10.1111/medu.14964

[CR13] Cooper, A., & Rodman, A. (2023). AI and Medical Education–a 21st Century Pandora’s Box. *New England Journal of Medicine*, *389*(5), 385–387. 37522417 10.1056/NEJMp2304993

[CR14] DiMaggio, P. J., & Powell, W. W. (1983). The iron cage revisited: Institutional isomorphism and collective rationality in organizational fields. *American Sociological Review,**48*, 147–160. 10.2307/2095101

[CR15] Evetts, J. (2013). Professionalism: Value and ideology. *Current Sociology*, *61*(5–6), 778–796. 10.1177/0011392113479316

[CR16] Fan, K. Y., Hu, R., & Singla, R. (2020). Introductory machine learning for medical students: A pilot. *Medical Education*, *54*(11), 1042–1043. 10.1111/medu.14318. 32862464 10.1111/medu.14318

[CR17] Fligstein, N., & McAdam, D. (2012). *A theory of fields*. Oxford University Press.

[CR18] Flyvbjerg, B. (2001). *Making social science matter: Why social inquiry fails and how it can succeed again*. Cambridge University Press.

[CR19] Freidson, E. (1988). *Profession of medicine: A study of the sociology of applied knowledge*. University of Chicago Press.

[CR20] French, M., & Miller, F. A. (2012). Leveraging the living laboratory: On the emergence of the entrepreneurial hospital. *Social Science and Medicine*, *75*(4), 717–724. 10.1016/j.socscimed.2012.04.010. 22627016 10.1016/j.socscimed.2012.04.010

[CR21] Gardner, J. (2022). Imaginaries of the data-driven hospital in a time of crisis. *Sociology of Health and Illness,**45*, 1–18. 10.1111/1467-9566.13592 10.1111/1467-9566.13592 36510787

[CR22] Gonzalo, J. D., Dekhtyar, M., Caverzagie, K. J., Grant, B. K., Herrine, S. K., Nussbaum, A. M., Tad, Y. D., White, E., & Wolpaw, D. R. (2021). The triple helix of clinical, research, and education missions in academic health centers: A qualitative study of diverse stakeholder perspectives. *Learning Health Systems*, *5*(4), e10250. 10.1002/lrh2.10250. 34667874 10.1002/lrh2.10250 PMC8512738

[CR23] Harish, V., Morgado, F., Stern, A. D., & Das, S. (2021). Artificial Intelligence and clinical decision making: The new nature of medical uncertainty. *Academic Medicine: Journal of the Association of American Medical Colleges*, *96*(1), 31–36. 10.1097/ACM.0000000000003707. 32852320 10.1097/ACM.0000000000003707

[CR24] Henwood, F., & Marent, B. (2019). Understanding digital health: Productive tensions at the intersection of sociology of health and science and technology studies. *Sociology of Health & Illness*, *41*(Suppl 1), 1–15. 10.1111/1467-9566.12898. 31599984 10.1111/1467-9566.12898

[CR25] Hodges, B. D. (2018). Learning from Dorothy Vaughan: Artificial intelligence and the health professions. *Medical Education*, *52*(1), 11–13. 10.1111/medu.13350. 28722148 10.1111/medu.13350

[CR26] Hoeyer, K. (2019). Data as promise: Reconfiguring Danish public health through personalized medicine. *Social Studies of Science*, *49*(4), 531–555. 31272287 10.1177/0306312719858697

[CR27] Hoeyer, K. (2023). *Data paradoxes: The politics of intensified data sourcing in contemporary healthcare*. MIT Press.

[CR28] Hoff, T. J., Pohl, H., & Bartfield, J. (2006). Teaching but not learning: How medical residency programs handle errors. *Journal of Organizational Behavior*, *27*(7), 869–896. http://www.jstor.org/stable/4093875. 10.1002/job.395

[CR29] Hsieh, H. F., & Shannon, S. E. (2005). Three approaches to qualitative content analysis. *Qualitative Health Research*, *15*(9), 1277–1288. 10.1177/1049732305276687. 16204405 10.1177/1049732305276687

[CR30] James, C. A., Wheelock, K. M., & Woolliscroft, J. O. (2021). Machine learning: The next paradigm shift in medical education. *Academic medicine: journal of the Association of American Medical Colleges*, *96*(7), 954–957. 10.1097/ACM.0000000000003943 (Comment in: Acad Med. 2021;96(9):1230 PMID: 34432660 [https://www.ncbi.nlm.nih.gov/pubmed/34432660] Comment in: Acad Med. 2021;96(9):1231 PMID: 34432661 [https://www.ncbi.nlm.nih.gov/pubmed/34432661]). 33496428

[CR31] Jasanoff, S. (2015a). Future imperfect: Science, technology, and the imaginations of modernity. In S. Jasanoff & S. H. Kim (Eds.), *Dreamscapes of modernity: Sociotechnical imaginaries and the fabrication of power* (pp. 1–33). University of Chicago Press.

[CR32] Jasanoff, S. (2015b). Imagined and invented worlds. In S. Jasanoff & S. H. Kim (Eds.), *Dreamscapes of modernity: Sociotechnical imaginaries and the fabrication of power* (pp. 321–341). University of Chicago Press.

[CR33] Johnston, S. C. (2018). Anticipating and training the physician of the future: The importance of caring in an age of artificial intelligence. *Academic Medicine: Journal of the Association of American Medical Colleges*, *93*(8), 1105–1106. 10.1097/ACM.0000000000002175. 29443717 10.1097/ACM.0000000000002175

[CR34] Karnieli-Miller, O., & Neufeld-Kroszynski, G. (2020). Combining machine learning and human reflective process for teaching communication skills. *Medical Education*, *54*(12), 1093–1095. 10.1111/medu.14391 (Comment on: Med Educ. 2020;54(12):1159–1170 PMID: 32776345 [https://www.ncbi.nlm.nih.gov/pubmed/32776345]). 33031599

[CR35] Katner, S. (2008). What is academic medicine? *Academic Medicine*, *83*(3), 205–206. 10.1097/ACM.0b013e318168e828

[CR36] Kenny, K., Broom, A., Page, A., Prainsack, B., Wakefield, C. E., Itchins, M., Lwin, Z., & Khasraw, M. (2021). A sociology of precision-in‐practice: The affective and temporal complexities of everyday clinical care. *Sociology of Health & Illness*, *43*(9), 2178–2195. 10.1111/1467-9566.13389. 34843108 10.1111/1467-9566.13389 PMC9299761

[CR37] Khurana, M. (2020). Keeping Pace: The need for digital health education in medical schools. *Academic medicine: journal of the Association of American Medical Colleges*, *95*(11), 1629–1630. 10.1097/ACM.0000000000003672 (Comment in: Acad Med. 2021;96(6):776 PMID: 34031299 [https://www.ncbi.nlm.nih.gov/pubmed/34031299]). 33109967

[CR38] Larson, M. (1977/2013). *The rise of professionalism: Monopolies of competence and sheltered markets* (2nd ed.). Transaction.

[CR39] Latour, B. (2007). *Reassembling the social: An introduction to actor-network-theory*. Oxford University Press.

[CR40] Lupton, D. (2012). M-health and health promotion: The digital cyborg and surveillance society. *Social Theory & Health*, *10*(3), 229–244. 10.1057/sth.2012.6. 10.1057/sth.2012.6

[CR41] Lupton, D. (2014). Beyond techno-utopia: Critical approaches to digital health technologies. *Societies*, *4*(4), 706–711. 10.3390/soc4040706. 10.3390/soc4040706

[CR42] Malterud, K., Siersma, V. D., & Guassora, A. D. (2016). Sample size in qualitative interview studies: Guided by information power. *Qualitative Health Research*, *26*(13), 1753–1760. 26613970 10.1177/1049732315617444

[CR43] Marent, B., & Henwood, F. (2023). Digital health: A sociomaterial approach. *Sociology of Health & Illness*, *45*(1), 37–53. 10.1111/1467-9566.13538. 36031756 10.1111/1467-9566.13538 PMC10088008

[CR44] McLoughlin, I., Garrety, K., & Wilson, R. (2017). *The digitalization of health care: Electronic records and the disruption of moral orders*. Oxford University Press.

[CR45] Merriam, S. B. (1988). *Qualitative research and case study applications in education*. Josey-Bass.

[CR46] Oh, H., Rizo, C., Enkin, M., & Jadad, A. (2005). What is eHealth (3): A systematic review of published definitions. *Journal of Medical Internet Research*, *7*(1), e1. 10.2196/jmir.7.1.e1. 15829471 10.2196/jmir.7.1.e1 PMC1550636

[CR47] Ovseiko, P. V., Davies, S. M., & Buchan, A. M. (2010). Organizational models of emerging academic health science centers in England. *Academic Medicine*, *85*(8), 1282–1289. 10.1097/ACM.0b013e3181e541bd. 20671453 10.1097/ACM.0b013e3181e541bd

[CR48] Porter, T. M. (1996). *Trust in numbers: The pursuit of objectivity in science and public life*. Princeton University Press. 10.1177/030631299029004007 11623934

[CR49] Presser, L. (2023). *Unsaid: Analyzing harmful silences*. Univeresity of California.

[CR50] Rapley, T. (2007). Exploring documents. *Doing conversation discourse and document analysis.* (pp. 112–124). SAGE Publications. 10.4135/9781849208901

[CR51] Reich, A. (2014). *Selling our souls: The commodification of hospital care in the United States*. Princeton University Press.

[CR52] Rowland, P., Anderson, M., Kumagai, A. K., McMillan, S., Sandhu, V. K., & Langlois, S. (2019). Patient involvement in health professionals’ education: A meta-narrative review [journal article]. *Advances in Health Sciences Education*, *24*(3), 595–617. 10.1007/s10459-018-9857-7. 30306292 10.1007/s10459-018-9857-7

[CR53] Rowland, P., Cupido, N., Albert, M., & Kitto, S. (2022). Morbidity and mortality rounds in the medical literature: A text based analysis of shifting knowledge regimes. *SSM - Qualitative Research in Health 2*. 10.1016/j.ssmqr.2022.100169. 10.1016/j.ssmqr.2022.100169

[CR54] Russell, R. G., Novak, L., Patel, L., Garvey, M., Craig, K. V., Jackson, K. J. T., Moore, G. P., D., & Miller, B. M. (2023). Competencies for the use of artificial intelligence-based tools by health care professionals. *Academic Medicine: Journal of the Association of American Medical Colleges*, *98*(3), 348–356. 10.1097/ACM.0000000000004963. 36731054 10.1097/ACM.0000000000004963

[CR55] Scott, W. R. (2005). Institutional theory: Contributing to a theoretical research program. *Great Minds in Management: The Process of Theory Development*, *37*, 460–484. 10.1093/oso/9780199276813.003.0022

[CR56] Smitherman, H. C. Jr., Baker, R. S., & Wilson, M. R. (2019). Socially accountable academic health centers: Pursuing a quadripartite mission. *Academic Medicine*, *94*(2), 176–181. 10.1097/ACM.0000000000002486. 30303815 10.1097/ACM.0000000000002486

[CR57] Spar, D. L. (2020). *Work mate marry love: How machines shape our human destiny*. Farrar, Straus and Giroux.

[CR58] Tavory, I., & Timmermans, S. (2019). Abductive analysis and grounded theory. In A. Bryant & K. Charmaz (Eds.), *The SAGE handbook of current developments in grounded theory* (pp. 532–546). USA: SAGE.

[CR59] Tavory, I., & Timmermans, S. (2014). *Abductive analysis: Theorizing qualitative research*. The University of Chicago.

[CR60] Tsoukas, H. (2018). Strategy and virtue- developing strategy-as-practice through virtue ethics. *Strategic Organization*, *16*(3), 323–351. 10.1177/1476127017733142

[CR61] Vaara, E. (2015). Critical discourse analysis as methodology in strategy as practice. In D. Golsorkhi, L. Rouleau, D. Seidl, & E. Vaara (Eds.), *Cambridge handbook of strategy as practice* (pp. 491–505). USA: Cambridge University Press.

[CR62] Wartman, S. A., & Combs, C. D. (2018). Medical education must move from the information age to the age of artificial intelligence. *Academic medicine: journal of the Association of American Medical Colleges*, *93*(8), 1107–1109. 10.1097/ACM.0000000000002044 (Comment in: Acad Med. 2019;94(3):300–301 PMID: 30817340 [https://www.ncbi.nlm.nih.gov/pubmed/30817340]). 29095704

